# [Corrigendum] Expression of cancerous inhibitor of protein phosphatase 2A in human triple negative breast cancer correlates with tumor survival, invasion and autophagy 

**DOI:** 10.3892/ol.2025.15047

**Published:** 2025-04-16

**Authors:** Shan Li, Ting-Ting Feng, Yang Guo, Xianjun Yu, Qiuyue Huang, Liang Zhang, Wei Tang, Ying Liu

Oncol Lett 12: 5370–5376, 2016; DOI: 10.3892/ol.2016.5374

Subsequently to the publication of the above paper, the authors have drawn the Editor's attention to the fact that they made inadvertent errors in assembling certain of the data shown in the colony formation assays in Fig. 2C on p. 5373 and the cell invasion assay data shown in Fig. 4A on p. 5374 (note that the data in question in Fig. 2C had previously appeared in the journal *Scientific Reports* in a paper that featured some of the same authors). However, they were able to consult their original data, and recognized how these errors occurred.

The revised versions of Figs. 2 and 4, showing alternative colony formation assay data from one of the repeated experiments in Fig. 2C and the correct cell invasion assay data for panels a and b in Fig. 4A, are shown on the next two pages. The authors regret the errors that were made during the compilation of the original figures, and are grateful to the editor of *Oncology Letters* for allowing them the opportunity to publish this Corrigendum. Note that the errors that were made in compiling this pair of figures did not have a significant impact on the conclusions reached in this study. All the authors agree with the publication of this corrigendum; furthermore, they apologize to the readership for any inconvenience caused.

## Figures and Tables

**Figure 2.**
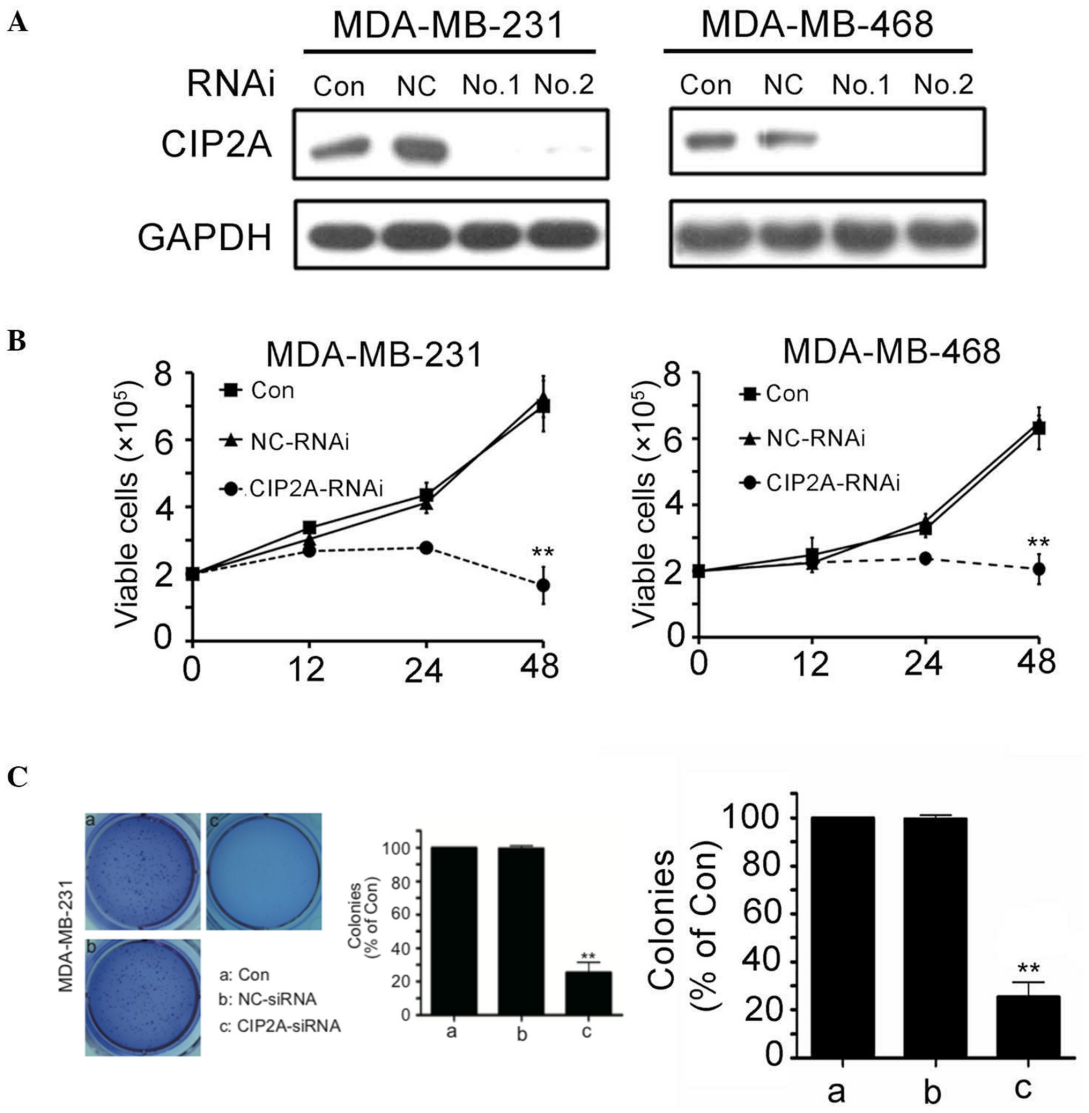
CIP2A depletion in triple negative breast cancer cell lines inhibits cell proliferation and clonogenic activity. (A) MDA-MB-231 and MDA-MB-468 cells were transfected with 100 nM CIP2A-specific siRNA1, siRNA2 or NC siRNA for 48 h, then cells were harvested for western blot analyses. (B) MDA-MB-231 and MDA-MB-468 cells were transfected with 100 nM CIP2A-specific siRNA or NC siRNA for 48 h. To evaluate cell growth, the cells were analyzed at indicated time points by trypan blue exclusion assay. (C) MDA-MB-231 cells were transfected with 100 nM CIP2A-specific siRNA or NC siRNA for 48 h. To evaluate cell colony formation, the cells were analyzed for 2 weeks by Soft-agar colony formation assay. **P<0.01. CIP2A, cancerous inhibitor of protein phosphatase 2A; Con, control; NC, negative control; RNAi, RNA interference..

**Figure 4.**
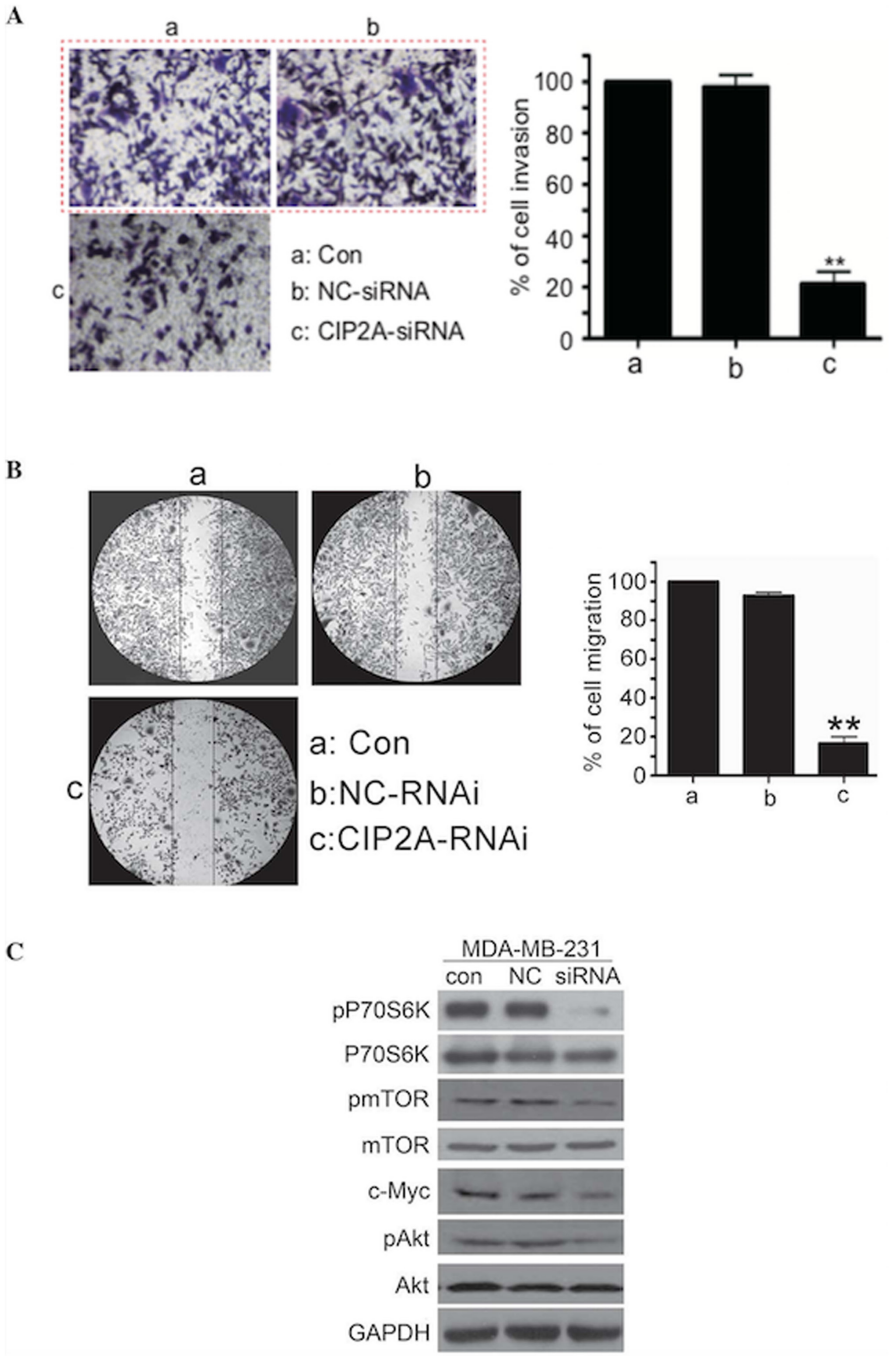
CIP2A depletion in triple negative breast cancer cells inhibits cell invasive behavior and Akt/mTOR/P70S6K phosphorylation. (A) MDA-MB-231 cells were transfected with 100 nM CIP2A-specific siRNA or NC siRNA for 48 h. To evaluate cell invasion, the cells were analyzed for 20 h by invasion assay. (B) MDA-MB-231 cells were transfected with 100 nM CIP2A-specific siRNA or NC siRNA for 48 h. To evaluate cell migration, the cells were analyzed for 24 h by wound healing assay. (C) MDA-MB-231 cells were transfected with 100 nM CIP2A-specific siRNA or NC siRNA for 48 h, then harvested for western blot analysis. con, control; CIP2A, cancerous inhibitor of protein phosphatase 2A; NC, negative control; RNAi, interfering RNA; siRNA, small interfering RNA; P70S6K, p70 ribosomal protein S6 kinase; pP70S6K, phosphorylated P70S6K; mTOR, mechanistic target of rapamycin; pmTOR, phosphorylated mTOR; pAkt, phosphorylated Akt.

